# Comparative studies of high-throughput biological graphs

**DOI:** 10.1186/1471-2105-12-S7-A12

**Published:** 2011-08-05

**Authors:** Jonathan Reyles, Charles Phillips

**Affiliations:** 1 Graduate School of Genome Science & Technology, UT-ORNL, Oak Ridge, TN, 37830, USA; 2 Department of Electrical Engineering and Computer Science, University of Tennessee, Knoxville, Knoxville, TN , 37996, USA

## Background

The exponential growth of biological data has given rise to new and difficult challenges. Because large data is often dealt with, it is inefficient to infer from each individual characteristics of a given dataset. Bioinformaticists are developing quantitative techniques to analyze and interpret key data properties. Graph algorithms can provide powerful and intuitive insight on such properties [1]. Using this approach, we collect biological data from transcriptomic and protein-protein interaction (PPI) sources. These data can be represented as a correlation matrix, where the rows are the vertices and the columns are the edges. We will analyze these graphs, and describe their differing structural characteristics.

## Materials and methods

We are using a high throughput method for graphical exploration of genomic and proteomic data. Experimental datasets are extracted from the public databases Biomart and Gene Expression Omnibus (GEO) [2,3]. R [4] and MATLAB are used to develop algorithms that compute and compare various structural characteristics. We specifically developed an in-house script used to output essential histograms and unweighted/weighted edges. We are currently developing protocols to analyze the comparison of transcriptomes and PPI sources.
